# Angiosuite-Based Awake Closed Reduction With Neurointerventional Standby for Cervical Fracture-Dislocation With Vertebral Artery Injury: A Technical Report

**DOI:** 10.7759/cureus.114816

**Published:** 2026-08-19

**Authors:** Junki Suzuki, Yasunori Sorimachi, Hiroyuki Sonoda, Hirotaka Chikuda

**Affiliations:** 1 Orthopaedic Surgery, Japanese Red Cross Maebashi Hospital, Maebashi, JPN; 2 Orthopaedic Surgery, Gunma University School of Medicine, Maebashi, JPN

**Keywords:** acute spinal trauma, angiography, awake reduction, cervical fracture-dislocation, vertebral artery injury

## Abstract

Cervical fracture-dislocation with vertebral artery injury (VAI) creates a treatment dilemma between urgent spinal realignment and cerebrovascular protection. We describe a hybrid workflow in which awake closed reduction is performed in the angiography suite with neurointerventional standby and report its feasibility in three patients. Between April 2023 and June 2024, patients with confirmed VAI underwent brain MRI and CT angiography (CTA), followed by transfer to the angiosuite. Arterial access and diagnostic angiography were performed before awake closed reduction under neurological monitoring. Endovascular rescue, including parent artery occlusion or thrombectomy, was available if neurological deterioration occurred. Closed reduction was successful in two patients, while one required intraoperative open reduction. One patient underwent pre-reduction coil embolization. These illustrative cases demonstrate the technical implementation and feasibility of the proposed workflow.

## Introduction

Traumatic cervical spine injuries may be associated with vertebral artery injury (VAI), particularly in patients with subaxial cervical fracture-dislocations [1,2]. It can cause posterior circulation infarction, yet the optimal treatment sequence is unsettled. Early closed reduction and decompression prevent secondary cord injury [3,4], but manipulation may aggravate VAI or dislodge thrombus [5]. Conversely, endovascular occlusion or reconstruction may stabilize the artery while delaying realignment and neurological recovery. Prior reports are heterogeneous and provide no consensus on sequencing [6,7].

We therefore implemented a workflow in which awake closed reduction is performed directly in the angiography suite with immediate neurointerventional standby. Unlike previously reported strategies that prioritize either spinal reduction or prophylactic endovascular treatment, our approach integrates awake reduction, continuous neurological monitoring, and immediate availability of endovascular rescue while minimizing delay to definitive spinal stabilization. This technical report describes the technical aspects of this workflow and illustrates its implementation using three illustrative cases.

## Technical report

Study design

This study was conducted in accordance with the principles of the Declaration of Helsinki. The study protocol was reviewed and approved by the Institutional Review Board of Japanese Red Cross Maebashi Hospital (approval no. 2026-02). The requirement for informed consent was waived due to the retrospective nature of the study. This retrospective technical report describes three illustrative cases treated using our institutional workflow. These cases are presented to demonstrate the technical implementation of the workflow rather than to evaluate clinical outcomes.

Patient selection and imaging

Candidates had traumatic cervical fracture-dislocation with radiologically confirmed VAI on CT angiography (CTA). Patients with hemodynamic instability or severe head injury precluding awake procedures were excluded. Preoperative brain MRI with diffusion-weighted imaging was performed to assess acute ischemia and collateral status. Injuries were classified according to the Allen and Ferguson (A&F) classification [8] and the Denver grading system [9].

Angiosuite setup and vascular access

**Figure 1:**
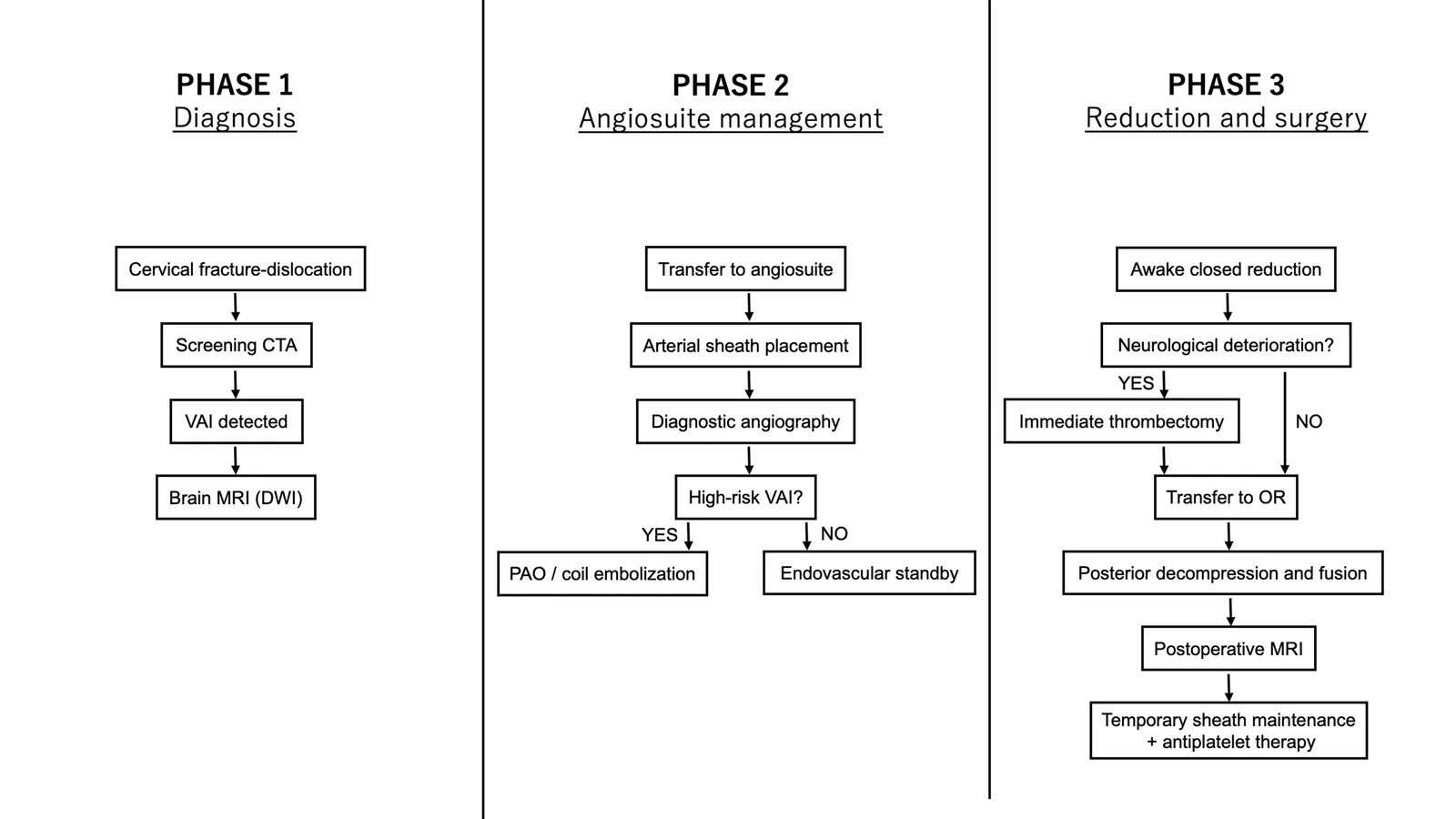
Institutional angiosuite-based management workflow for cervical fracture-dislocation with VAI VAI: Vertebral artery injury; CTA: Computed tomography angiography; DWI: Diffusion-weighted imaging; PAO: Parent artery occlusion; OR: Operating room

After VAI confirmation on CTA, patients were transferred to the angiosuite. The neurointerventional team established arterial access and placed a vascular sheath. Diagnostic angiography was performed to assess injury characteristics and collateral flow. The decision to perform prophylactic endovascular treatment before reduction was individualized following a multidisciplinary discussion between the spine and neurointerventional teams, based on angiographic findings and the overall clinical situation. No predefined treatment algorithm was used. If deemed necessary, preventive endovascular treatment, coil embolization, or parent artery occlusion (PAO) was considered before reduction. Otherwise, endovascular rescue devices were prepared. The overall management workflow used in our institution is summarized in Figure 1.

Awake reduction technique

Patients were positioned supine with the head secured in a halo ring. The primary spine surgeon stood at the head of the table and performed manual traction through the halo ring under continuous fluoroscopic guidance. Cervical alignment was adjusted according to the fracture morphology, with flexion and rotational maneuvers applied as needed to facilitate reduction. Two assistants stabilized the patient’s torso to provide countertraction. Neurological status was continuously assessed by repeated evaluation of motor function in the upper and lower extremities, sensory changes, level of consciousness, and patient-reported neurological symptoms throughout the procedure. Analgesia or light sedation was administered selectively according to the patient’s condition while preserving the ability to perform continuous neurological examination. If neurological deterioration occurred, endovascular rescue (mechanical thrombectomy or PAO) was to be initiated immediately through the pre-positioned sheath. The sheath was temporarily maintained after reduction.

Surgery and postoperative care

After attempted closed reduction in the angiosuite, patients were transferred directly to the operating room for posterior decompression and instrumented fusion. When closed reduction failed, open reduction was performed intraoperatively before fixation. Early postoperative brain MRI was obtained to detect new cerebral infarctions. Patients were managed in the intensive care unit, and the arterial sheath was removed after several days if no clinical or radiological evidence of cerebral ischemia emerged. Oral antiplatelet therapy and vascular imaging follow-up (CTA or MR angiography) were arranged in collaboration with the neurointerventional team.

Illustrative cases

Case One

**Figure 2:**
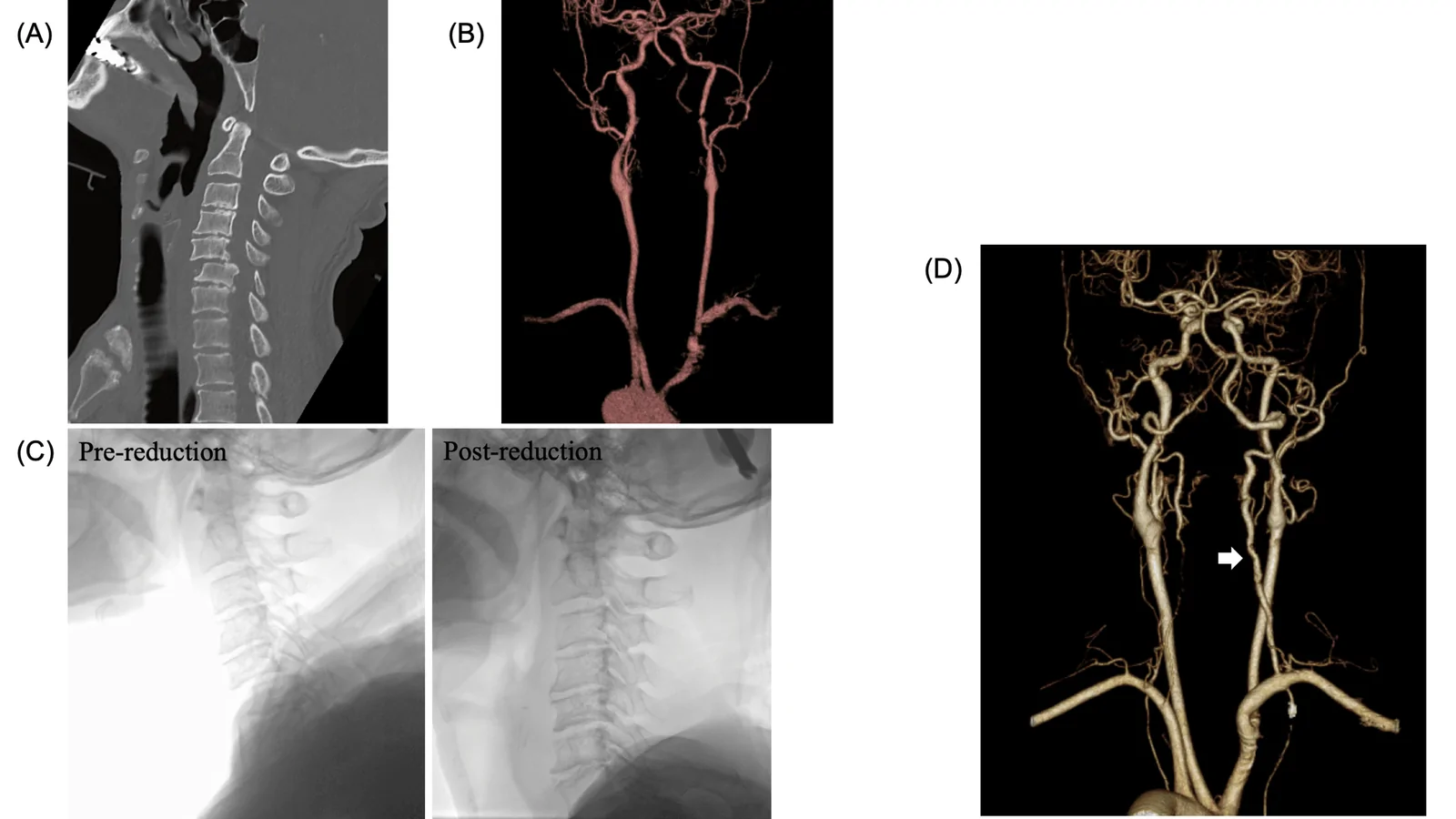
Imaging findings of case one (A) Sagittal CT showing C5-6 cervical fracture-dislocation; (B) Pre-reduction CTA demonstrating bilateral vertebral artery occlusion; (C) Fluoroscopic images obtained before and after awake closed reduction; (D) Follow-up CTA demonstrating recanalization of the right vertebral artery (arrow) CTA: Computed tomography angiography; VAI: Vertebral artery injury

A 78-year-old man sustained a high-energy fall while cutting trees, resulting in a C5-6 fracture-dislocation. Representative imaging findings for this case are shown in Figure 2. The CTA demonstrated bilateral VAI, and brain MRI revealed an asymptomatic cerebellar infarction. On admission, he presented with complete tetraplegia (American Spinal Injury Association Impairment Scale (AIS) grade A). In the angiosuite, an arterial sheath was inserted, and diagnostic angiography confirmed preserved basilar artery flow via collateral circulation. Awake closed reduction using halo ring traction was successfully achieved. No new neurological deficits occurred. The patient then underwent posterior decompression and C5-7 instrumented fusion. Neurological function improved to AIS C without ischemic complications, and follow-up CTA demonstrated right vertebral artery recanalization.

Case Two

A 30-year-old man suffered a C3-4 fracture-dislocation after a trampoline fall. The CTA revealed right-sided VAI without cerebral infarction; his initial neurological status was AIS grade C. In the angiosuite, preventive coil embolization was selected after multidisciplinary discussion based on the diagnostic angiographic assessment and the overall clinical situation. Reduction was successful, and neurological function improved to AIS grade E without ischemic complications.

*Case Three* 

A 47-year-old man experienced a C4-5 fracture-dislocation due to blunt trauma at work. The CTA showed left-sided VAI, and the MRI revealed no acute infarction. On admission, he was AIS grade A. Awake closed reduction in the angiosuite was attempted but unsuccessful; however, no neurological deterioration occurred. Open reduction and posterior fusion were subsequently performed, and neurological function improved to AIS grade C without stroke. A summary of the patient characteristics, treatments, and outcomes of all three cases is provided in Table 1.

**Table 1:** Summary of clinical characteristics and outcomes of the three patients with cervical fracture-dislocation and VAI A&F: Allen and Ferguson classification; VAI: Vertebral artery injury; AIS: American Spinal Injury Association Impairment Scale

Case no.	Age/sex	Injury mechanism	Injury level (A&F type)	VAI side/Denver grade	Initial AIS	Reduction method	Endovascular treatment	Surgical procedure	Injury-to-reduction time	Operative time	Neurological outcome (final AIS grade)	Posterior circulation stroke	Hospital stay (days)
1	78/M	Fall from height (tree cutting)	C5-6 (flexion-distraction)	Bilateral/Ⅳ (occlusion)	A	Closed (awake)	None	Posterior decompression and C5-7 fusion	6 hours	3 hours 10 min	C	None	87
2	30/M	Trampoline accident	C3-4 (flexion-distraction)	Right/Ⅳ (occlusion)	C	Closed (awake)	Coil embolization (pre-reduction)	Posterior decompression and fusion	3 hours 30 min	3 hours 38 min	E	None	42
3	47/M	Workplace blunt trauma	C4-5 (flexion-distraction)	Left/Ⅳ (occlusion)	A	Failed closed, converted to open	None	Posterior decompression and fusion	5 hours 30 min	3 hours 43 min	C	None	65

## Discussion

The optimal management strategy for cervical fracture-dislocation associated with VAI remains controversial. A key clinical dilemma is whether urgent spinal realignment should be prioritized over cerebrovascular protection. Early reduction is important to minimize secondary spinal cord injury [3,4]; however, manipulation of an unstable cervical spine may exacerbate vascular injury or provoke embolic posterior circulation stroke.

Several reports have described ischemic complications occurring after cervical reduction in patients with VAI. Tumialán and Theodore reported a fatal basilar artery thrombosis following reduction of cervical spondyloptosis [10]. Similarly, Nakao et al. described embolic cerebral infarction after reduction in a patient with traumatic vertebral artery occlusion [11]. These reports highlight the potential risk of vascular complications associated with spinal realignment maneuvers.

Conversely, performing prophylactic endovascular treatment before reduction may stabilize the injured vessel but can delay spinal decompression and neurological recovery. Previous studies have described preventive PAO or coil embolization before reduction [12,13], but such strategies may not always be necessary and may prolong the time to spinal realignment.

**Figure 3:**
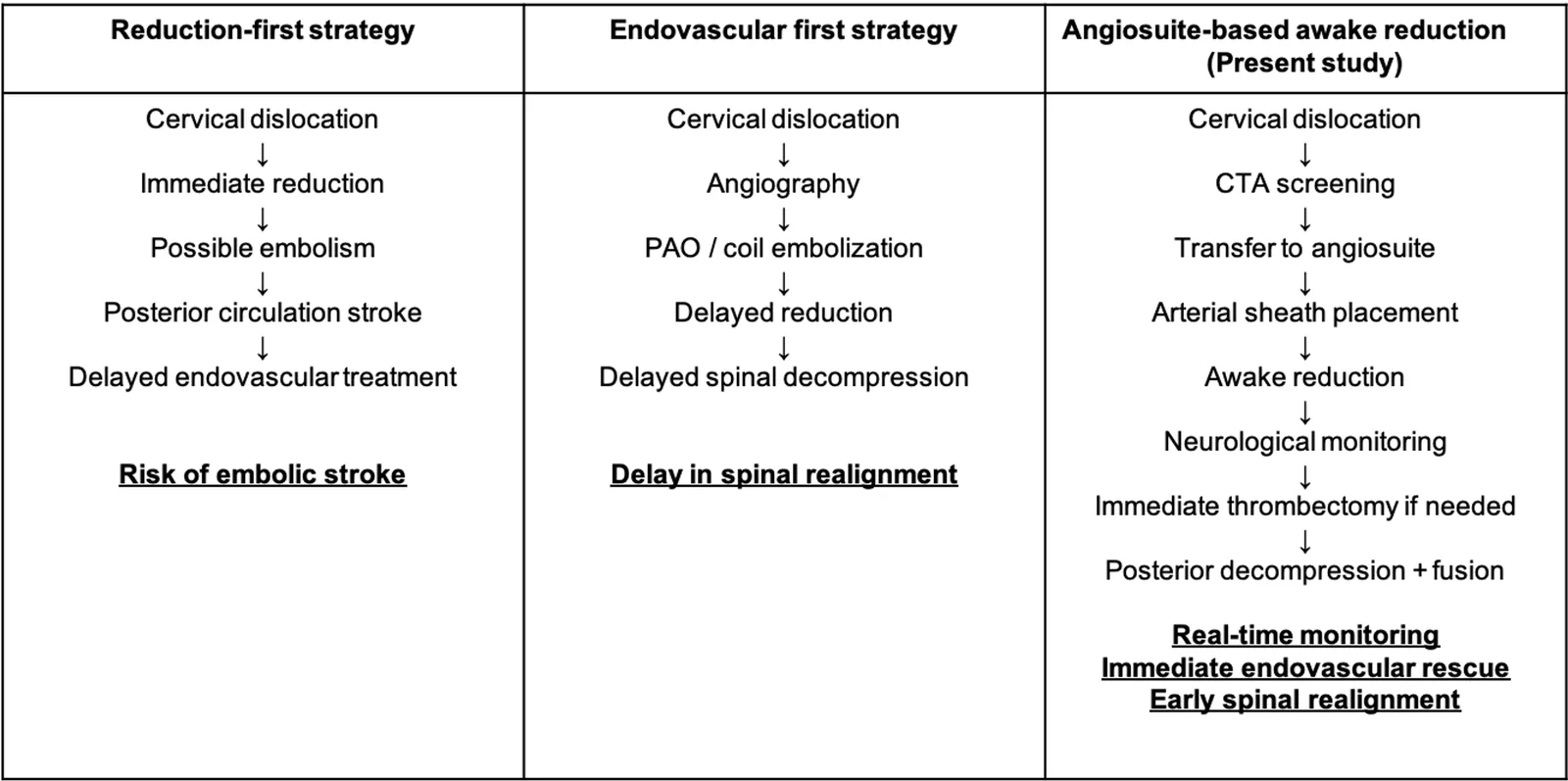
Comparison of management strategies for cervical fracture-dislocation with VAI The conventional reduction-first strategy carries a potential risk of embolic posterior circulation stroke. The endovascular-first strategy may delay spinal realignment and decompression. The proposed angiosuite-based awake reduction workflow combines early spinal realignment with continuous neurological monitoring and immediate availability of endovascular rescue when neurological deterioration occurs. VAI: Vertebral artery injury; PAO: Parent artery occlusion; CTA: Computed tomography angiography

Our workflow was designed to address this unresolved sequencing dilemma. The conceptual differences between conventional strategies and the angiosuite-based workflow are illustrated in Figure 3. By performing awake closed reduction in the angiosuite with neurointerventional standby, real-time neurological monitoring can be achieved while maintaining immediate access to endovascular rescue therapy.

The primary purpose of this workflow is to create an environment in which cervical reduction can be performed while maintaining continuous neurological assessment and immediate access to neurointerventional treatment if neurological deterioration occurs. The technique requires close coordination between spine surgeons, neurointerventionalists, anesthesiologists, and radiology staff, as well as prior preparation of fluoroscopy, vascular access, and rescue devices.

Awake reduction allows immediate recognition of neurological symptoms that may indicate posterior circulation ischemia, including altered consciousness, diplopia, dysarthria, limb weakness, or worsening sensory deficits. In addition, securing arterial access before reduction enables immediate endovascular rescue if vascular complications occur. Mechanical thrombectomy may be lifesaving when embolic occlusion develops during reduction [14,15]. Consequently, performing the reduction in the angiosuite allows rapid transition to thrombectomy or PAO if neurological deterioration is detected. In the present illustrative cases, implementation of this workflow did not appear to delay definitive surgery substantially. Furthermore, delayed thromboembolic events have been reported after blunt cerebrovascular injury, with stroke occurring several days after trauma [16]. Accordingly, short-term sheath maintenance in the early postoperative period provides a practical safety net against delayed ischemic stroke.

No patient required emergent endovascular rescue, although its availability provided an important safety net. The protocol also allowed individualized management of vascular injuries, as demonstrated by the case in which preventive coil embolization was performed prior to reduction.

Appropriate patient selection is essential for successful implementation of this workflow. Based on these illustrative cases, this workflow may be particularly suitable for hemodynamically stable patients with subaxial cervical fracture-dislocation and radiologically confirmed VAI who can undergo continuous neurological assessment during reduction. Conversely, patients with severe traumatic brain injury, inability to undergo serial neurological examination, profound hemodynamic instability, or the need for immediate life-saving surgery are poor candidates for this workflow because awake neurological monitoring cannot be reliably performed. 

This study has several limitations. First, the sample size was small, reflecting the rarity of this specific injury combination. Second, the absence of ischemic complications in this series may be influenced by case selection bias. Third, the proposed workflow requires the availability of an angiosuite and a neurointerventional team, which may not be feasible in all institutions. We acknowledge that this workflow requires immediate access to an angiosuite and an experienced neurointerventional team and therefore may be most applicable in tertiary referral centers. Institutions without these resources should individualize treatment according to local expertise. Institutions wishing to adopt this workflow should establish predefined collaboration between spine surgeons and neurointerventional teams and ensure immediate access to an angiography suite before attempting implementation.

To our knowledge, this is the first technical report describing an angiosuite-based workflow integrating awake closed reduction, continuous neurological monitoring, and immediate neurointerventional standby for cervical fracture-dislocation associated with VAI. These cases illustrate the technical feasibility of implementing the proposed workflow in selected patients under appropriate institutional conditions. However, the present study was limited to three cases, and further multicenter studies are needed to validate its reproducibility and clinical utility.

## Conclusions

We describe an angiosuite-based awake closed reduction workflow with immediate neurointerventional standby for cervical fracture-dislocation with VAI. In the illustrative cases presented in this report, the proposed workflow appeared feasible and allowed continuous neurological assessment while maintaining immediate access to endovascular rescue when necessary. Although further studies are needed to validate its generalizability, this approach may represent a practical option for managing this challenging clinical scenario.

## References

[1] Temperley HC, McDonnell JM, O'Sullivan NJ, Waters C, Cunniffe G, Darwish S, Butler JS (2023). The incidence, characteristics and outcomes of vertebral artery injury associated with cervical spine trauma: a systematic review. Global Spine J.

[2] Willis BK, Greiner F, Orrison WW, Benzel EC (1994). The incidence of vertebral artery injury after midcervical spine fracture or subluxation. Neurosurgery.

[3] Newton D, England M, Doll H, Gardner BP (2011). The case for early treatment of dislocations of the cervical spine with cord involvement sustained playing rugby. J Bone Joint Surg Br.

[4] Oae K, Kamei N, Sawano M, Yahata T, Morii H, Adachi N, Inokuchi K (2023). Immediate closed reduction technique for cervical spine dislocations. Asian Spine J.

[5] Indo M, Oya S, Shojima M, Inokuchi K, Yahata T, Sugiyama S, Matsui T (2019). Prevention of thromboembolic infarction after surgery for traumatic cervical fracture with vertebral artery occlusion by preoperative endovascular coil embolization. World Neurosurg.

[6] Merrill S, Clifton W, Valero-Moreno F, Damon A, Rahmathulla G (2020). Vertebral artery injury with coinciding unstable cervical spine trauma: mechanisms, evidence-based management, and treatment options. Cureus.

[7] Zygogiannis K, Benetos IS, Evangelopoulos DS, Koulalis D, Pneumaticos SG (2024). Blunt traumatic vertebral artery injury after cervical fracture dislocation: a systematic review of the literature. Cureus.

[8] Allen BL Jr, Ferguson RL, Lehmann TR, O'Brien RP (1982). A mechanistic classification of closed, indirect fractures and dislocations of the lower cervical spine. Spine.

[9] Biffl WL, Moore EE, Offner PJ, Brega KE, Franciose RJ, Burch JM (1999). Blunt carotid arterial injuries: implications of a new grading scale. J Trauma.

[10] Tumialán LM, Theodore N (2012). Basilar artery thrombosis after reduction of cervical spondyloptosis: a cautionary report. J Neurosurg Spine.

[11] Nakao Y, Terai H (2014). Embolic brain infarction related to posttraumatic occlusion of vertebral artery resulting from cervical spine injury: a case report. J Med Case Rep.

[12] Suga Y, Mitome-Mishima Y, Yoshida K, Higo T, Nishioka K, Oishi H (2022). Evaluation for vertebral artery injury with cervical dislocated fracture and optimal treatment before reduction. J Neuroendovasc Ther.

[13] Nakamura Y, Kusakabe K, Nakao S, Hagihara Y, Matsuoka T (2021). Vertebral artery occlusion associated with blunt traumatic cervical spine injury. Acute Med Surg.

[14] Goyal M, Menon BK, van Zwam WH (2016). Endovascular thrombectomy after large-vessel ischaemic stroke: a meta-analysis of individual patient data from five randomised trials. Lancet.

[15] Hirota S, Takahashi S, Yoshimura M (2023). Mechanical thrombectomy and parent artery occlusion for acute basilar artery occlusion due to vertebral fracture and artery dissection: a case report. J Neuroendovasc Ther.

[16] Cothren CC, Biffl WL, Moore EE, Kashuk JL, Johnson JL (2009). Treatment for blunt cerebrovascular injuries: equivalence of anticoagulation and antiplatelet agents. Arch Surg.

